# Unexpected Hazards with Dental High Speed Drill

**DOI:** 10.3390/dj5010010

**Published:** 2017-01-25

**Authors:** Kassahun Hailu, David Lawoyin, Alison Glascoe, Andrea Jackson

**Affiliations:** Howard University School of Dentistry, Washington, DC 20059, USA; dlawoyin@howard.edu (D.L.); aglascoe@howard.edu (A.G.); ajackson@howard.edu (A.J.)

**Keywords:** high-speed, Bur, accident, prevention

## Abstract

An expected accident can happen at any time during a routine practice in the dental office due to the types of instruments used. One of the instruments used in routine dental practice is a high speed drill and a bur. If Personal Protective Equipment (PPE) is not practiced at any time in the dental office, very serious injuries could easily happen to the clinician, staff or to the patient.

## 1. Introduction

Work-related injuries can happen anywhere and anytime. They are unexpected occurrences and can affect anyone, regardless of their background, skillset, or professional affiliation. The injury can be minor or life-threatening. It is important to be aware of the types of injuries that people can sustain in the work place as well as being knowledgeable about how to prevent them from occurring [1,2].

Dental professionals are more vulnerable to work place injuries during day-to-day operations in the office as compared to any other healthcare professionals. According to research on work-related injuries, most occupational hazard injuries in the dental office cause the dentist to become more cautious about his overall health [1]. Due to the nature of the dental profession, the chances of being exposed to dental materials that may cause a simple allergic reaction or result in a systemic disease, are understood and noted by the professional [3]. Prolonged and static postural positions are also commonplace in the dental profession. These types of positions may also result in musculoskeletal and nerve injuries of the neck, shoulder, back and hands [4].

Despite technological advancements in the dental profession, work-related injuries are inevitable. Many of these injuries occur as a direct result of needle sticks, which result in concern about contracting blood borne infections. Injuries of a projectile nature may also occur. For example, a small sharp bur from the high speed can cause direct bodily injury from hitting any part of the body, such as the eye. Another possible injury could occur from oral surgery instruments during treatment. An injury can happen at any time, to the clinician, staff, or patient during patient treatment, due to the nature of instrument design. Some of the instruments are sharp, such as drill burs. In addition, hand instruments with sharp working ends can also cause injury during treatment. These kinds of instruments are very prone to possibly causing damage to the clinician or the patient.

Studies have shown that a dental practitioner may have a wide variety of physical and psychological ailments, which are induced or aggravated by work specifically and greatly affects the health of dental professional [5].

The selection of appropriate dental instruments such as high-speed hand piece and a bur is key for the safe and effective removal of dental hard tissues and caries in an efficient manner that also maximizes ergonomics for the dentist.

## 2. Case Study

This is a case of a dentist with 16 years of experience who performed an examination on a new patient who presented with a dental emergency. One of the patient’s chief complaints was “I cannot bite evenly and I always hit my right side first. I keep getting pain in my lower jaw every time I chew.” Based on the patient’s chief complaint, a limited clinical exam was done. The clinical examination revealed the presence of three metal ceramic crowns on the mandibular right posterior on teeth numbers: 28, 29 and 30. These crowns appeared to have been placed recently. The occlusal anatomy was flat with no evidence of porcelain fracture. The margins seemed to be questionable due to a slight catch with an explorer. The surrounding soft and hard tissue appeared to be with in normal limits. There was no swelling, bleeding, or inflammation. After checking patient’s occlusion, the clinical findings were consistent with hyper-occlusion in the areas of teeth numbers 28, 29, and 30, which were covered with PFM crowns. The patient was informed that the two crowns on the mandibular right side showed evidence of being slightly higher than the rest of the teeth and required adjustment.

At the commencement of the adjustment procedure, the dentist noticed a slight vibration and a funny noise coming from the hand piece. He stopped the flushing process and checked to see if the bur was engaged properly. He decided to try flushing the hand piece one more time while sitting at the 12 o’clock position. After stepping on the rheostat, he heard an unusual sound from the high-speed hand piece again. Then, suddenly, a piece from the diamond bur split and flew off. At the exact same time, he felt a slight sting on his own chin but felt no pain. Within a few seconds, he noticed a small amount of blood dripping from the mask he was wearing. He removed the mask in order to check his chin in the mirror and saw a very small bleeding laceration. The bleeding was managed by rinsing the area with hydrogen peroxide (H_2_O_2_) and applying a band-aid. The entire ordeal lasted between 5 and 10 min and the patient was unaware of what had happened.

This patient was the last patient of the day, and thus the dentist presumed that the bur, which flew out of the hand-piece, might have been bent from previous use, which may have caused the problem. The dentist placed a new diamond bur into the hand-piece and completed the procedure. The patient was dismissed in stable condition. The dentist then went back and began palpating his chin in the area of the injury. He noticed a slight swelling as one would expect. He tried to locate the piece of the bur in the surrounding area but was unable to do so. He left the office and went home without finding the broken piece of the bur.

**Figure 1:**
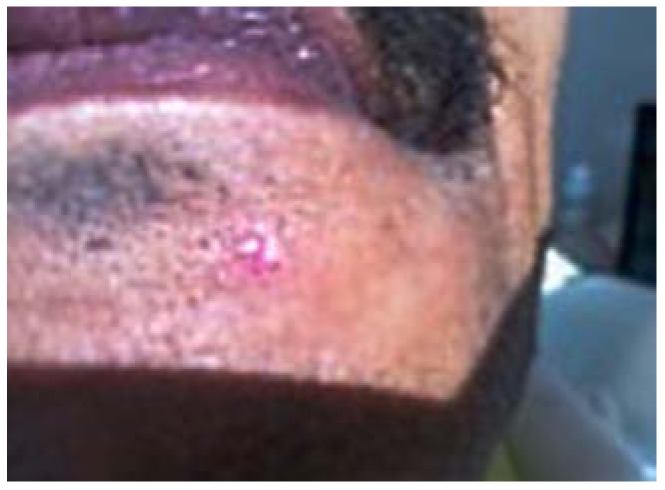
24 h Pre-op extra-oral mild swelling.

**Figure 2:**
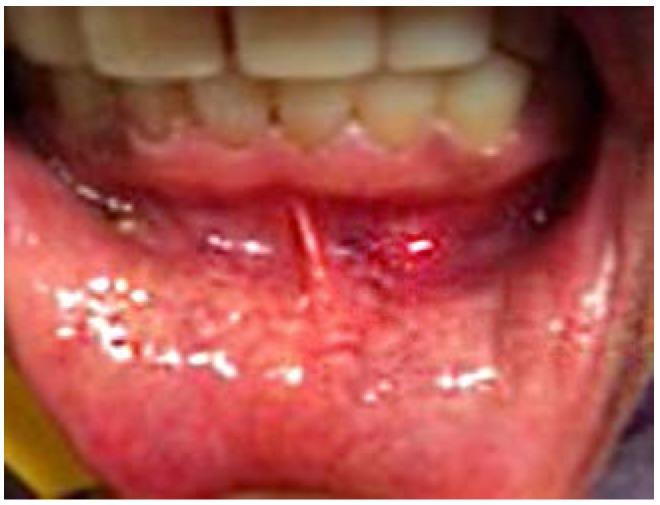
24 h Pre-op intra-oral mild swelling.

**Figure 3:**
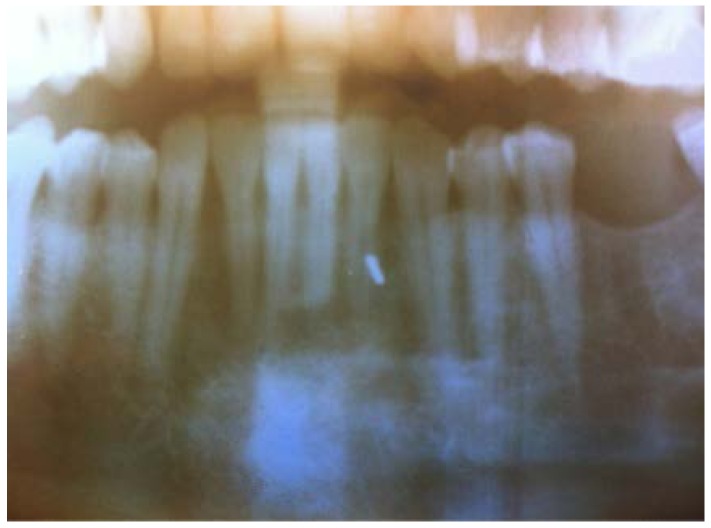
Panoramic X-ray.

**Figure 4:**
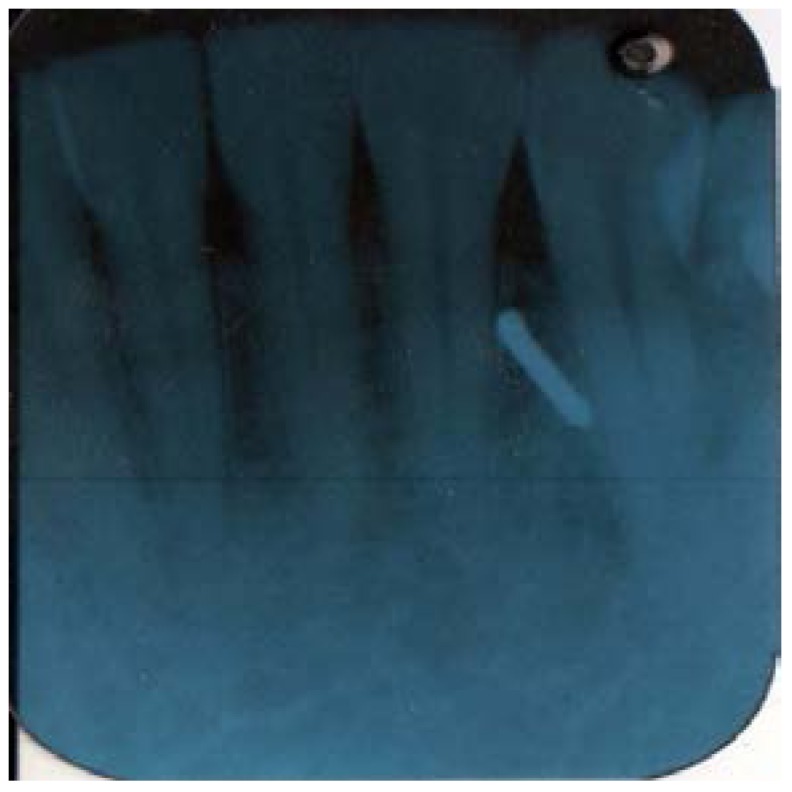
Pri-apical X-ray.

Later in the day, the dentist felt a slight hardness and slight pain upon palpitating the area. By the next day, the swelling had progressed slightly and the dentist felt a slight pain upon palpitation or any movement, such as chewing Figure 1 and Figure 2. He did not think that the small piece of bur was lodged in his chin. A decision was made to have radiographs taken, which included a panoramic and a peri-apical radiograph. Subsequent review of the radiographs revealed a radiopaque image consistent with a broken piece of the bur in the soft tissue of his chin, in the vestibule of the mandibular anterior left side Figure 3 and Figure 4.

**Figure 5:**
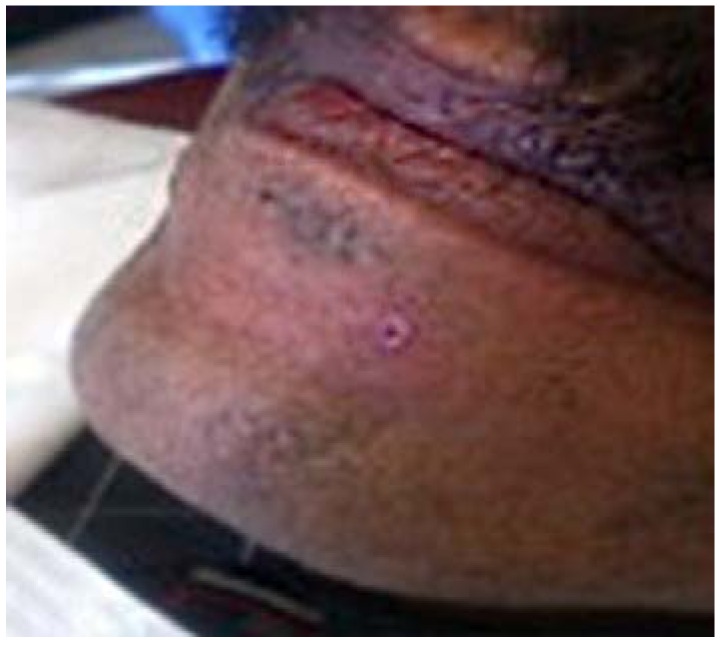
72h Post-op extra-oral swelling reduced.

**Figure 6:**
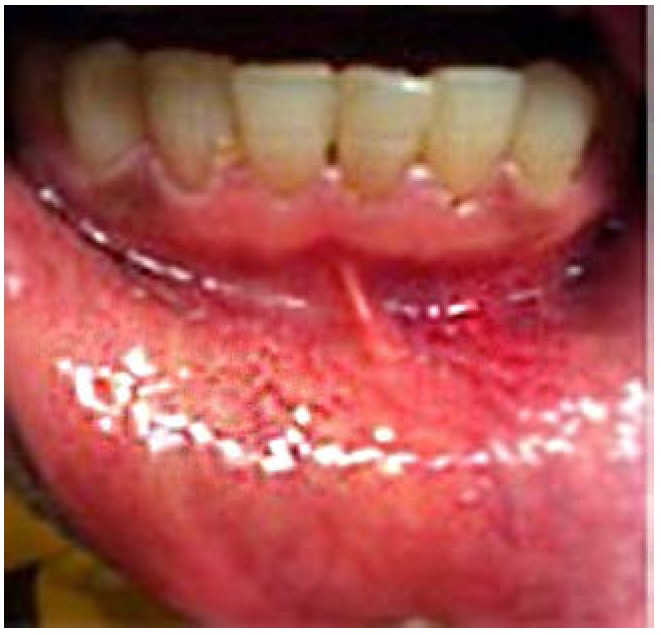
72 h post-op extra-oral swelling reduced.

After identifying the problem, an initial attempt to remove the foreign object in the body was aborted due to pain. A later attempt was made by a colleague using local anesthesia. This resulted in the successful removal of the piece of bur without any complication. Figure 5 and Figure 6 post treatment showed complete healing.

## 3. Discussion

Most dentists are more concerned about their patients and staff and pay little attention to the potential for injury to themselves. Unexpected occupational injuries can occur in dental practices, despite the state of the arts equipment in the office. These hazards can be broadly categorized as: physical, chemical, biological, mechanical, and psychological. Physical and mechanical hazards can include such things as eye injuries resulting from projectiles, cuts from sharp instruments, or puncture wounds from needles or other sharps. Such injuries can result in the transmission of serious infectious diseases to the dental worker as well [6].

Review of the literature on dental tool manufacturing guidelines, how each tool is made, and their safety concerns perhaps could minimize dental office accidents by a great percentage. The dental bur is one of the tools frequently used in the dental office. Each type has its own unique design and is used for a specific purpose. Eric Schwarzenbach, president of Rollmatic Inc., Mundelein, IL, USA, agreed that tool support is vital when grinding. “We have a system where we clamp the bur right behind the head” he said. “A dental bur is very weak at the shank, and if you don’t have a good support system, you can’t grind accurately and it will deflect all the time” [7,8,9].

Although most dental burs have the joint located in the posterior part of the head, others have the joint located within the shank and have carbide necks and heads. A carbide bur is stiffer and stronger than a steel bur, but it is also more brittle. A carbide bur’s neck if subjected to a sudden blow or shock may fracture, whereas a steel bur’s neck tends to bend. A bur that is even slightly bent produces increased vibration and overcutting that causes unnecessary damage to tooth structure. Although steel necks reduce the risk of fracture during use, they may cause severe problems if bent. Either type can be satisfactory, and other design factors are varied to take maximal advantage of the properties of the material used [10,11].

Dental practitioners must educate themselves and be familiar with every tool they use in the office by following the manufacturer’s safety guidelines and instructions. A replacement guideline for each instrument is one of the most important instructions necessary to follow in order to avoid accident or injury to the patient or staff during any dental procedure. It is understandable that following the recommended guidelines may increase the overhead cost for the office; however, the benefits far outweigh the costs when safety for the patients, staff and doctor is the primary consideration.

It is advisable, and in some instances mandatory, to practice using items such as rubber dams, ligatures, or throat packs, during dental procedures in order to protect the patient. Additionally, these procedural practices may also be considered proper personal protective equipment or Personal Protective Equipment (PPE) in order to protect the patient, staff and clinician. Even when following all precautions, accidents may still happen and when they do occur, one must make sure to take total control of the situation and assume the worse when a dental item disappears during treatment. Knowing what to do can be extremely important, both medically and legally [12].

## 4. Conclusions

Every member of the dental team must be aware of safety precautions and how to practice prevention, which is a necessity for the safety of the patients and themselves, regardless whether the treatment is simple or complex. The OSHA, Occupational Safety and Health Administration, guidelines for Personal Protective Equipment (PPE) must always be adhered to and followed strictly.

The general health of the dentist and staff in the dental office is a priority matter in order to create a safe working environment and a sound practice. Following safety guidelines can minimize most of the dental related injuries that happen in the dental office. There is a need therefore to educate and make dentists aware of potential hazards and the methods of their prevention. We have an ethical obligation that prevention must come first when we perform any type of treatment to the patient.

## References

[1] Ayatollahi J., Ayatollahi F., Ardekani A.M., Bahrololoomi R., Ayatollahi J., Ayatollahi A., Owlia M.B. (2012). Occupational hazards to dental staff. Dent. Res. J..

[2] Kanoff J.M., Turalba A.V., Andreoli M.T., Andreoli C.M. (2010). Characteristics and outcomes of work-related open globe injuries. Am. J. Ophthalmol..

[3] Puriene A., Janulyt V., Musteikyte M., Bendinskaite R. (2007). General Health of Dentists. Literature review. Stomatologija.

[4] Abhishek M., Gupta M., Upadhyaya N. (2013). Status of occupational hazards and their prevention among dental professionals in Chandigarh, India: A comprehensive questionnaire survey. Dent. Res. J..

[5] Hanson K. (2012). Taking a bite: Dental bur manufacturing. Micro Manuf..

[6] Little D. www.dentalofficemag.com.

[7] Roberson T., Heymann H., Swift E., Sturdevant C. (2013). Sturdevant’s Art and Science of Operative Dentistry.

[8] History of Handpieces. http://dentalhandpiecesrepairs.com/Historyofdentalhandpieces.aspx.

[9] Robel B., Hill E.E. (2008). A practical review of prevention and management of ingested/aspirated dental items. Gen Dent..

[10] Kasloff Z. (1964). Enamel cracks caused by rotary instruments. J. Prosthet. Dent..

[11] Puriene A., Janulyte V., Musteikyte M., Bendinskaite R. (2007). General Health of Dentists. Literature Review. Stomatologija Baltic Dental Maxillofacial. J..

[12] Rytkönen Esorainen E. (2001). Vibration of Dental Handpieces. AIHAJ.

